# Wallenberg Syndrome: Two Case Reports Highlighting Vertebral Artery Dissection and Thrombophilia as Distinct Etiologies

**DOI:** 10.7759/cureus.100355

**Published:** 2025-12-29

**Authors:** Sara Remelhe Sá, Rita Pera, João Lagarteira, Lilia Castelo Branco, Cristiana Batouxas

**Affiliations:** 1 Internal Medicine Department, Unidade Local de Saúde do Nordeste, Bragança, PRT

**Keywords:** brainstem infarction, neurovascular imaging, posterior circulation stroke, thrombophilia, vertebral artery dissection, wallenberg syndrome

## Abstract

Wallenberg syndrome, or lateral medullary syndrome, is caused by ischemia of the posterolateral medulla, most often attributed to vertebral artery or posterior inferior cerebellar artery occlusion. The condition is characterized by heterogeneous sensory, cerebellar, and autonomic deficits induced by damage to multiple brainstem nuclei and tracts. Herein, we report two cases of Wallenberg syndrome associated with different etiological mechanisms. Case 1 involved a 55-year-old man with vertigo, diplopia, anisocoria with ipsilateral ptosis, and left-sided sensory deficits. Magnetic resonance imaging (MRI) revealed a 9-mm diffusion-restricted lesion in the right posterolateral medulla and signs of proximal intracranial vertebral artery dissection. Case 2 involved a 41-year-old man with vertigo, dysphonia, dysphagia, ipsilateral facial sensory loss, and contralateral body hypoalgesia. MRI showed an acute small infarct in the right posterolateral medulla without evidence of dissection. The computed tomography scan findings were inconclusive in both cases, thereby emphasizing the diagnostic value of diffusion-weighted MRI. The cardiac studies had unremarkable results. Both patients received antiplatelet therapy, vascular risk factor management, and early rehabilitation. These cases underscore the diversity of mechanisms underlying Wallenberg syndrome. Further, they emphasize the importance of prompt diagnosis, vascular imaging, and multidisciplinary management to improve outcomes.

## Introduction

Wallenberg syndrome, or lateral medullary syndrome, is caused by ischemia of the posterolateral medulla, most commonly attributed to vertebral artery or posterior inferior cerebellar artery occlusion. Blood flow interruption affects key structures including the nucleus ambiguus, vestibular nuclei, spinal trigeminal nucleus, spinothalamic tract, and descending sympathetic fibers. Hence, patients present with characteristic motor, sensory, and autonomic deficits [1].

In older patients with vascular risk factors such as hypertension, diabetes, dyslipidemia, and smoking, the most common etiology is atherosclerotic vertebral artery disease [2]. In younger individuals, vertebral artery dissection is a significant cause of brainstem stroke, and it may occur spontaneously or after minor cervical trauma [3].

Herein, we present two cases of Wallenberg syndrome with distinct etiologies - one secondary to vertebral artery dissection and the other potentially related to thrombophilia without evidence of dissection.

## Case presentation

Case 1

A 55-year-old male construction worker with a long history of smoking and alcohol consumption presented with sudden-onset vertigo, imbalance, headache, and diplopia. The neurological examination revealed anisocoria with right-sided miosis and ptosis, left-sided hypesthesia in the upper and lower limbs, and gait instability. The laboratory tests, including inflammatory markers and coagulation parameters, had unremarkable findings. Non-contrast-enhanced head computed tomography (CT) scan showed no acute ischemia or hemorrhage.

**Figure 1:**
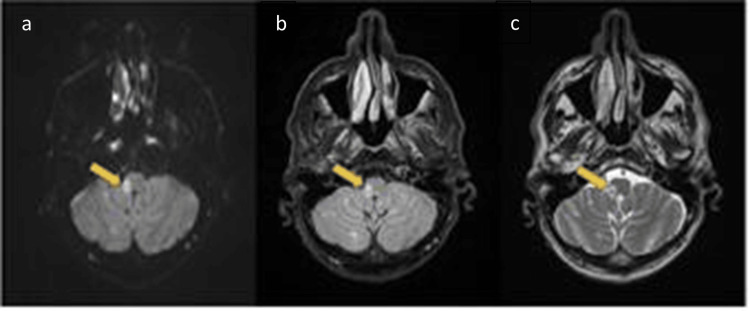
Brain magnetic resonance imaging showed signs of proximal intracranial right vertebral artery dissection with approximately 50% luminal reduction visible. Diffusion-weighted (a), fluid-attenuated inversion recovery (b), and T2-weighted (c) imaging sequences.

Magnetic resonance imaging (MRI) identified a 9-mm diffusion-restricted focus in the right posterolateral medulla consistent with acute ischemia. Angio-MRI showed signs of proximal intracranial right vertebral artery dissection with approximately 50% luminal reduction (Figure 1).

Electrocardiogram, echocardiogram, and 24-hour Holter monitoring revealed no arrhythmias. Antiplatelet monotherapy was initiated, and the patient was referred for follow-up at the Internal Medicine department.

Case 2

A 41-year-old man without a previous remarkable medical history, particularly head/neck trauma, presented to the emergency department due to headache, vertigo, dysphonia, dysphagia, loss of balance with gait instability, impaired taste sensation, and loss of pain and temperature sensation on the right face and contralateral body, five hours of evolution, but with preserved muscle strength. The neurological examination revealed right-sided ptosis, horizontal nystagmus, impaired temperature and pain sensation in the right side of the face and contralateral body, and leftward deviation of the uvula. The laboratory test revealed an activated protein C resistance and Factor V Leiden (FVL) mutation (Table 1).

**Table 1:** Laboratory test results ANA, antinuclear antibodies; ANCA, antineutrophil cytoplasmic antibody

Parameters	Results	Normal range
Protein C (%)	54	70 – 140
Protein S (%)	116	74.1 – 146.1
Protein C resistance	1.33	> 2.1 = negative
Factor V Leiden mutation	Positive	-
Antithrombin III (%)	102	83 – 128
Lupus anticoagulant	1.14	< 1.2 = negative
Anticardiolipin antibodies (IgG, IgM)	< 0.9, 1.7	< 10 = negative
ANA antibodies (UI/mL)	0.1	< 0.7 = negative
ANCA antibodies (PR3/MPO) (UI/mL)	0.1	< 2.0 = negative

**Figure 2:**
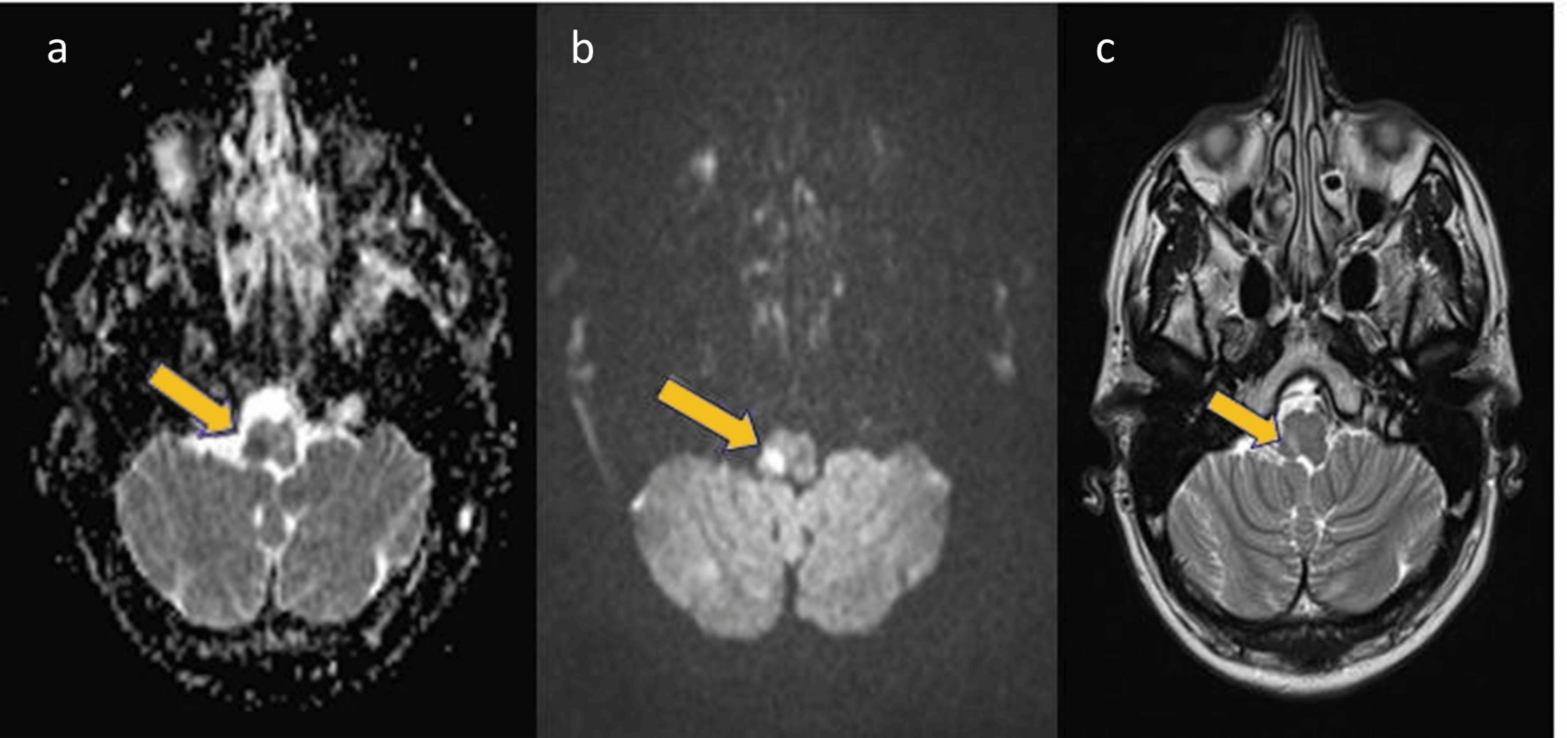
Brain magnetic resonance imaging showed an infra-centimetric acute ischemic lesion in the right posterolateral medulla. Diffusion-weighted (a), fluid-attenuated inversion recovery (b), and T2-weighted (c) imaging sequences.

​​The head CT scan finding was inconclusive. MRI showed an infra-centimetric acute ischemic lesion in the right posterolateral medulla, consistent with Wallenberg syndrome, without evidence of vertebral artery dissection (Figure 2).

Atrial fibrillation was ruled out based on the electrocardiogram, echocardiogram, and Holter monitoring findings. The treatment included blood pressure optimization, antiplatelet therapy, statin for secondary prevention, deep vein thrombosis prophylaxis, and early rehabilitation.

## Discussion

These two cases illustrate the clinical and etiological heterogeneity of Wallenberg syndrome, thereby emphasizing the need for a high index of suspicion in patients presenting with dissociated sensory loss, oculomotor abnormalities, dysphagia, vertigo, or ipsilateral Horner syndrome. The classic symptom constellation reflects the involvement of the spinothalamic tract, spinal trigeminal nucleus, nucleus ambiguus, vestibular nuclei, inferior cerebellar peduncle, and descending sympathetic fibers, all of which are vulnerable to ischemia in the lateral medulla [1,2].

Atherosclerotic occlusion of the vertebral artery or posterior inferior cerebellar artery is the most common cause of lateral medullary infarction [2]. However, vertebral artery dissection is increasingly recognized as a major etiology in younger and middle-aged adults, accounting for up to 25% of posterior circulation strokes in this population [3]. Case 1 involved vertebral artery dissection with luminal narrowing, thereby emphasizing the importance of early vascular imaging - preferably magnetic resonance angiography or computed tomography angiography - particularly if symptoms follow head/neck movement or if traditional cardiovascular risk factors are absent [3,4].

Case 2 presented with a thrombophilic condition, FVL, previously associated with an increased risk of venous thromboembolism [5]. Although its role in posterior circulation stroke is not well defined, isolated reports and reviews suggest a potential association in selected cases [6,7]. The presence of activated protein C resistance indicates a possible prothrombotic substrate, supporting the hypothesis that hypercoagulability may have contributed to ischemia in the absence of vascular dissection or cardioembolic sources.

In both cases, non-contrast-enhanced CT scan did not identify acute abnormalities, consistent with literature demonstrating its low sensitivity for detecting posterior fossa ischemia due to beam-hardening artifacts and the complex anatomy of the medulla [8,9]. Diffusion-weighted MRI remains the diagnostic gold standard, as it can identify small infarcts with high sensitivity [1].

Management with antiplatelet therapy, secondary stroke prevention measures, and early rehabilitation is in accordance with current evidence-based recommendations. The choice between antiplatelet therapy and anticoagulation in vertebral artery dissection remains controversial; however, randomized and observational studies have demonstrated no significant difference in preventing recurrent ischemic events, supporting antiplatelet therapy as a reasonable first-line option [10]. Early multidisciplinary rehabilitation is essential for addressing dysphagia, gait instability, and vestibular symptoms, significantly improving functional outcomes [11].

Overall, these cases reinforce the broad etiological spectrum of Wallenberg syndrome and highlight the importance of prompt neuroimaging, vascular assessment, and individualized management strategies.

## Conclusions

In this study, the two cases showed diverse mechanisms underlying Wallenberg syndrome, which included vertebral artery dissection and hypercoagulability associated with FVL mutation. Despite the different etiologies, both cases exhibited the classical clinical phenotype and underscored the diagnostic limitations of CT scan, with MRI serving as the definitive modality for validating medullary ischemia. Comprehensive etiological evaluation, including vascular imaging and thrombophilia screening in specific patients, is essential for obtaining an accurate diagnosis and facilitating targeted treatment. The early initiation of antiplatelet therapy, risk-factor management, and structured rehabilitation remain the fundamental components of care. Knowledge of atypical etiologies, such as arterial dissection and inherited thrombophilia, may improve diagnostic accuracy and contribute to better functional outcomes in patients with lateral medullary infarction.
